# Development of *Burkholderia mallei* and *pseudomallei* vaccines

**DOI:** 10.3389/fcimb.2013.00010

**Published:** 2013-03-11

**Authors:** Ediane B. Silva, Steven W. Dow

**Affiliations:** Department of Microbiology, Immunology, and Pathology, Regional Center of Excellence in Emerging Diseases and Bioterrorism, Colorado State UniversityFt. Collins, CO, USA

**Keywords:** *Burkholderia*, glanders, melioidosis, immune response, vaccine, live attenuated

## Abstract

*Burkholderia mallei* and *Burkholderia pseudomallei* are Gram-negative bacteria that cause glanders and melioidosis, respectively. Inhalational infection with either organism can result in severe and rapidly fatal pneumonia. Inoculation by the oral and cutaneous routes can also produce infection. Chronic infection may develop after recovery from acute infection with both agents, and control of infection with antibiotics requires prolonged treatment. Symptoms for both meliodosis and glanders are non-specific, making diagnosis difficult. *B. pseudomallei* can be located in the environment, but in the host, *B. mallei* and *B. psedomallei* are intracellular organisms, and infection results in similar immune responses to both agents. Effective early innate immune responses are critical to controlling the early phase of the infection. Innate immune signaling molecules such as TLR, NOD, MyD88, and pro-inflammatory cytokines such as IFN-γ and TNF-α play key roles in regulating control of infection. Neutrophils and monocytes are critical cells in the early infection for both microorganisms. Both monocytes and macrophages are necessary for limiting dissemination of *B. pseudomallei*. In contrast, the role of adaptive immune responses in controlling *Burkholderia* infection is less well understood. However, T cell responses are critical for vaccine protection from *Burkholderia* infection. At present, effective vaccines for prevention of glanders or meliodosis have not been developed, although recently development of *Burkholderia* vaccines has received renewed attention. This review will summarize current and past approaches to develop *B. mallei* and *B. pseudomalllei* vaccines, with emphasis on immune mechanisms of protection and the challenges facing the field. At present, immunization with live attenuated bacteria provides the most effective and durable immunity, and it is important therefore to understand the immune correlates of protection induced by live attenuated vaccines. Subunit vaccines have typically provided less robust immunity, but are safer to administer to a wider variety of people, including immune compromised individuals because they do not reactivate or cause disease. The challenges facing *B. mallei* and *B. pseudomalllei* vaccine development include identification of broadly protective antigens, design of efficient vaccine delivery and adjuvant systems, and a better understanding of the correlates of protection from both acute and chronic infection.

## Introduction: diseases caused by *B. mallei* and *B. pseudomallei* infection

Glanders is the clinical disease caused by *Burkholderia mallei* infection. Horses, mules, and donkeys are highly susceptible to this bacterium and these infected animals serve as a natural reservoir, since the organism is not able to survive in the environment alone. Equine glanders transmission occurs though ingestion or direct contact with fomites. These zoonotic infections occur by contact with infected animals or following direct contact with *B. mallei* bacterial cultures (Dvorak and Spickler, 2008). The symptoms of glanders depend upon the route of infection and are often characterized by pneumonia, septicemia, and chronic suppurative infections of the skin (Srinivasan et al., 2001; Anuntagool and Sirisinha, 2002; Rosenbloom et al., 2002).

*B. mallei* infection spreads primarily hematogenously, and studies in hamster infection models have demonstrated lesions in the liver, spleen and lungs 6 h after intraperitoneal (i.p.) inoculation (Fritz et al., 1999). The host immune response to *B. mallei* depends critically on activation of innate immune responses (Goodyear et al., 2010, 2012b), whereas there is limited knowledge regarding adaptive immune responses during glanders infection.

Melioidosis is caused by infection with *Burkholderia pseudomallei* and the agent can cause infection in humans and animals (Cheng and Currie, 2005). The disease can develop following subcutaneous (s.c.) inoculation or ingestion or inhalation of the bacterium (Wiersinga and Van Der Poll, 2009). Certain environmental conditions can also increase the risk of contracting melioidosis. For example the rainy season may be associated with increased risk of septic and pneumonic forms of melioidosis (Currie and Jacups, 2003). As is the case with glanders, the route of *B. pseudomallei* infection also contributes to the severity of the disease (Barnes and Ketheesan, 2005).

*B. pseudomallei* infect a variety of cells, including mononuclear phagocytes and some non-phagocytic cells. Once inside macrophages, the organism can escape endocytic vesicles and live free within the cytoplasm (Jones et al., 1996). *B. mallei* and *B pseudomallei* use BimA, a protein required for actin-based motility (Stevens et al., 2005b) and BimA homologuous proteins for cell-to-cell spread (Stevens et al., 2005a; Allwood et al., 2011) and the formation of multi-nucleate giant cells (Harley et al., 1998). *In vivo*, the bacterium can spread hematogenously to the liver, spleen or brain, and can also produce sepsis. However, the events underlying the spread of *B. pseudomallei* and the apparent predilection for sites such as the brain remain poorly understood (White, 2003; Owen et al., 2009). The olfactory sensory nerve is the main route to melioidosis brain infection (and to some degree the trigeminal nerve), though the authors could not explain the mechanism of travel of *B. pseudomallei* via the nerves (Owen et al., 2009).

The development of symptoms of melioidosis is dependent on several factors, including bacterial strain differences, variations in the host immune response, and the route of infection (Cheng and Currie, 2005). Acute infection is associated with severe pneumonia or rapid septicemias (Chaowagul et al., 1989). In the chronic stages of infection, meliodosis can lead to the formation of abscesses in multiple organs, or can lead to asymptomatic infection, which can apparently be reactivated after many years (Ngauy et al., 2005). Recent studies by our group suggest that the site of asymptomatic infection may actually be the gastrointestinal tract (Goodyear et al., 2012a).

Currently both *B. pseudomallei* and *B. mallei* are considered as potential bioweapons and they are classified by the Centers for Disease Control as category B select agents (Dance, 2002; Warawa and Woods, 2002). The use of *B. mallei* and *B. pseudomallei* as bioweapon stems primarily from the fact that clinical disease can develop following inhalational exposure to even very low infective doses of the organisms. In addition, *B. pseudomallei* is also endemic in the soil and water of certain regions of the world, adding to the ease with which bacterial strains can be isolated and propagated (Currie and Jacups, 2003; Peacock, 2006; Gilad et al., 2007; Ko et al., 2007). The *B. pseudomallei* organism can also be easily manipulated genetically (Mack and Titball, 1996). Effective treatment of melioidosis is limited by inherent bacterial resistance to several different classes of antibiotics and treatment of acute illness is typically associated with high morbidity and mortality rates (Warawa and Woods, 2002). For all the reasons noted above, development of effective *B. mallei* and *B. pseudomallei* vaccines are a priority in the emerging infectious disease field.

## Host immune response to *Burkholderia* infection

Clinical and experimental studies indicate that *Burkholderia* infection rapidly activates the innate immune system. Depending on the dose and route of infection, the organism can be rapidly eliminated by this early innate immune response, primarily through the actions of monocytes and natural killer (NK) cells. Macrophages play a critical role in the early phase of infection by controlling organism replication (Breitbach et al., 2006). However, if the initial infection is not controlled and chronic infection is established, CD4^+^ T cells become more important for long-term control of infection (Haque et al., 2006b). By determining what is required for a successful immune response against acute or chronic *Burkholderia* infection, it may also be possible to elucidate what is required for successful vaccine-induced protective immunity.

### Innate immune responses to *Burkholderia* infection

A fully functional innate immune system is critical to the efficient, early control of *Burkholderia* infection. The innate immune response to *Burkholderia* has been investigated in much greater detail than the adaptive immune response. Several key cell types are activated following *Burkholderia* infection, along with their cell surface signaling molecules, including Toll-like receptors (TLRs), NOD-like receptors (NLRs) and caspases. Key cytokines including MCP-1, INF-γ, and TNF-α also are required for protective immunity to *Burkholderia* infection.

#### Role of TLR signaling in *Burkholderia* infection

Cell surface expression of TLR1, TLR2, and TLR4 is upregulated on monocytes and neutrophils from patients infected with *B. pseudomallei* (Wiersinga et al., 2007a). Unlike the case with many Gram-negative pathogens, the lipopolysaccharide (LPS) from *B. pseudomallei* appears to activate macrophages only through TLR2 and not via TLR4 (Wiersinga et al., 2007b). Thus, in *B. pseudomallei* infection, TLR2 can be stimulated directly, whereas activation of TLR4 requires the coreceptors CD14 and MD2 (Hii et al., 2008). Mice lacking the TLR2 receptor are actually protected from *B. pseudomallei* infection, whereas TLR4 defective mice do not exhibit increased susceptibility to either *B. mallei* or *B. pseudomallei* infection (Wiersinga et al., 2007b; Goodyear et al., 2012b). Macrophage activation by *B. pseudomallei* results in production of pro-inflammatory cytokines, including TNF-α, IL-1β, and IL-6 (West et al., 2008). Host susceptibility to melioidosis is modulated by TLR polymorphisms, but the mechanisms of this effect are not well understood. For example: TLR4 polymorphisms are associated with increased risk of melioidosis and TLR6-1-10 region single nucleotide polymorphisms in diabetes patients are associated with differential susceptibility to melioidosis compared with other illnesses (West et al., 2012).

#### NLRs and *Burkholderia* infection

The NLRs NOD1 or NOD2 regulate IL-18 production following *B. pseudomallei* infection (Hii et al., 2008). Production of IL-18 in response to inflammasome signaling was shown to be protective against *B. pseudomallei* infection (Wiersinga et al., 2007c). Mice deficient in inflammasome components including ASC, caspase-1, NLRC4, and NLRP3 were more susceptible to *B. pseudomallei* infection than wild type animals (Ceballos-Olvera et al., 2011).

#### MyD88 signaling

MyD88 signaling plays a key role in the protection against infection with both *B. pseudomallei* and *B. mallei*. For example, MyD88^−/−^ mice infected with either *B. pseudomallei* or *B. mallei* were much more susceptible to infection than wild type mice (Wiersinga et al., 2008; Goodyear et al., 2012b). Interestingly, in MyD88^−/−^ mice infected with *B. pseudomallei*, there was reduced early pulmonary neutrophil recruitment and a diminished activation of neutrophils compared to wild type mice (Wiersinga et al., 2008). The MyD88-dependent pathway may also contribute to a detrimental host inflammatory response (Ventura et al., 2009). MyD88^−/−^ mice infected with *B. mallei* showed a decreased inflammatory response (Goodyear et al., 2012b). For example, numbers of monocytes, myeloid dendritic cells (DC) and neutrophils in the lungs of *B. mallei* infected MyD88^−/−^ mice were reduced compared to wild type animals. The key defect in the MyD88^−/−^ mice infected with *B. mallei* appeared to be a lack of IFN-γ production, as IFN-γ production was completely absent in MyD88^−/−^ animals and treatment of MyD88^−/−^ animals with rIFN-γ partially restored protective immunity (Goodyear et al., 2012b).

#### Role of Caspase-1 in *Burkholderia* infection

*B. pseudomallei* infection was shown to induce caspase-1 activation, causing macrophage cell death and triggering the release of IL-1β and IL-18 (Sun et al., 2005). The caspase-1 pathway was also shown to regulate IFN-γ production following *B. pseudomallei* infection. Caspase-1^−/−^ mice were highly susceptible to *B. pseudomallei* infection with high bacterial loads and greatly diminished production of IFN-γ (Breitbach et al., 2009).

#### Role of MCP-1 in *Burkholderia* infection

Monocyte chemoattractant protein-1 (MCP-1) was shown recently to play a critical role in protective immunity to *B. mallei* respiratory infection (Goodyear et al., 2010). Both MCP-1^−/−^ mice and mice lacking the MCP-1 receptor (CCR2^−/−^ mice) were more susceptible to *B. mallei* infection, with significantly higher bacterial burdens in lung, liver and spleen 3 days following intranasal challenge compared to wild type animals. Lack of monocyte and inflammatory DC recruitment to the lungs appeared to be the primary defect in MCP-1^−/−^ animals, and in fact these animals actually had significantly greater numbers of neutrophils in the lungs. In addition, IFN-γ concentrations were markedly decreased in infected MCP-1^−/−^ and CCR2^−/−^ mice. Treatment with exogenous rIFN-γ helped restore effective immunity and increased survival significantly (Goodyear et al., 2010).

#### Role of TNF-α

TNF-α is required to control the infection caused by *B. pseudomallei* in mouse models. For example, TNF-α^−/−^ mice were much more susceptible to *B. pseudomallei* infection, with increased bacterial loads in spleen and liver and high mortality rates of TNF-α^−/−^, TNFR1, and TNFR2^−/−^ animals (Barnes et al., 2008). However, in human melioidosis patients with sepsis, increased TNF-α production is thought to actually be associated with higher mortality rates (Suputtamongkol et al., 1992). While BALB/c and C57Bl/6 mice both readily develop chronic infection, BALB/c mice are more susceptible to acute infection (Liu et al., 2002). There are some notable differences in the immune response to *B. pseudomallei* between mouse strains. For example, TNF-α concentrations were markedly higher in the livers of BALB/c mice following lethal *B. pseudomallei* infection, whereas concentrations were lower in the livers of C57Bl/6 mice (Ulett et al., 2000). Thus, TNF-α appears to play a dual role in regulation of both protection and susceptibility to *Burkholderia* infection. It would therefore be difficult to predict whether vaccine induction of TNF-α production would be a desirable biological endpoint.

#### Role of IFN-γ

Unlike TNF-α, the role of IFN-γ in protection from *Burkholderia* infection is unambiguous and IFN-γ is obviously a key protective cytokine. For example, IFN-γ^−/−^ mice are highly susceptible to infection with both *B. mallei* and *B. pseudomallei* (Santanirand et al., 1999; Haque et al., 2006b; Koo and Gan, 2006; Rowland et al., 2006; Easton et al., 2007; Goodyear et al., 2009, 2010). In addition, mice depleted of IFN-γ with antibodies are also very susceptible to infection (Santanirand et al., 1999; Breitbach et al., 2006; Haque et al., 2006b; Whitlock et al., 2008; Wiersinga et al., 2008). IFN-γ production regulates intracellular killing of *Burkholderia* and organ burdens are uniformly higher in IFN-γ^−/−^ mice compared to wild type animals infected with *B. pseudomallei*. The source of IFN-γ production *in vivo* has not been fully elucidated, but our studies have shown high levels of IFN-γ production by NK cells in the lungs (Goodyear et al., 2009). *In vitro* studies have shown that *B. pseudomallei* infection elicits IFN-γ production from NK cells, NKT cells, and conventional T cells in an IL-12 and IL-18-dependent manner (Haque et al., 2006b).

#### Role of IL-12

The presence of both subunits of IL-12 (IL-12p40 and IL-12p35) was shown to be critical for IFN-γ production *in vitro* in response to heat-killed *B. mallei* (Rowland et al., 2006). In addition, neutralization of IL-12 *in vivo* resulted in increased susceptibility of mice to lethal infection (Santanirand et al., 1999). The role of IL-12 may be primarily to drive NK cells and T cells to IFN-γ production.

### Innate cellular immunity to *Burkholderia* infection

#### Role of macrophages

Macrophages play a critical role in early stages of *B. pseudomallei* infection, as deduced from studies in C57BL/6 and BALB/c mice (Breitbach et al., 2006). Macrophage killing of *Burkholderia* is dependent on the presence of IFN-γ derived from NK cells, CD8^+^, and TCR-γδ T cells (Rowland et al., 2006). Though controversial, there is also evidence that iNOS is also required for macrophage control of *B. pseudomallei* infection, though *in vitro* and *in vivo* experiments give conflicting results in this regard (Utaisincharoen et al., 2004; Breitbach et al., 2006, 2011). Macrophage dysfunction in diabetes mellitus is thought to explain in part the extreme susceptibility of diabetics to lethal meliodosis infection (Hodgson et al., 2011).

#### Role of monocytes

Recent studies performed in our laboratory indicate that monocytes play a critical role in the early control of *B. mallei* infection (Goodyear et al., 2010). For example, mice in which monocyte migration is impaired (CCR2^−/−^ mice) are extremely susceptible to inhaled and parenteral challenge with *B. mallei*. This increased susceptibility is associated with significant impairment in monocyte and DC entry into the lungs following inhalational challenge with *B. mallei*. In these mice, production of IL-12 by inflammatory DC is also significantly impaired following *B. mallei* challenge. In another study, depletion of monocytes using a depleting antibody to Gr-1 also increased susceptibility to *B. pseudomallei* infection (Rowland et al., 2010).

#### Role of neutrophils

Gr-1^+^ neutrophils are also essential to early control of *B. mallei* infection, based on the results of antibody depletion studies (Rowland et al., 2010). Neutrophil depletion with a cross-reactive Gr-1 antibody severely exacerbated disease, resulting in an acute lethal infection associated with a 1000-fold increase in lung bacterial loads within 4 days. Neutrophils also play an important indirect role in the generation of the early cytokine environment in the lungs (Easton et al., 2007). Neutrophil dysfunction may also help explain the increased susceptibility of diabetics to meliodosis, as polymorphonuclear (PMN) cell responses to *B. pseudomallei* were impaired due to defects in migration and apoptosis (Chanchamroen et al., 2009).

#### Role of natural killer cells

NK cells are estimated to produce up to 80% of IFN-γ in the early stages of *B. pseudomallei* infection and IFN-γ is a key cytokine for control of *B. pseudomallei* infection (Haque et al., 2006b). NK cells are also the major source of IFN-γ production in early *B. mallei* infection (Rowland et al., 2006; Goodyear et al., 2012b). Thus, NK cells may help control *Burkholderia* infection indirectly by activating macrophages via IFN-γ to kill intracellular *Burkholderia*.

### Adaptive immune response to *Burkholderia*

While the role of innate immunity in controlling both *B. mallei* and *B. pseudomallei* infections has been investigated in relative detail, much less is known about the role of adaptive immune responses (T cell and B cell responses) in controlling *Burkholderia* infection.

#### Role of T cells

Protection against *B. pseudomallei* infection appears to depend on both strong humoral and cell-mediated immunity (Healey et al., 2005). For example, CD4^+^ T cells were shown in mouse infection models to play an important role in resistance to *B. pseudomallei* infection during the later stages of the acute infection (Haque et al., 2006b). In contrast, the same authors found that CD8^+^ T cells were much less important in resistance to melioidosis (Haque et al., 2006b). However, in general very little is known regarding the role of specific T cells subsets in regulating the tempo or the course of *Burkholderia* infection.

#### Role of B cells and antibodies

IgM antibodies were detected only in patients with localized *B. pseudomallei* infection. On the other hand, IgG antibodies were detected in patients during the acute infection. IgG1 and IgG2 antibodies were observed in all patient groups while an IgG3 response was seen only in survivors of septicaemic infection, suggesting that these antibody isotypes may be a good indicator of active disease (Ho et al., 1997) or a good monitor of therapy (Vasu et al., 2003). The level of IgA, IgG, and IgM titers increase during an active infection with glanders, but the titers decreased within 14 months after infection (Waag et al., 2012).

Serum from meliododis patients contains antibodies that target LPS (Bryan et al., 1994; Ho et al., 1997; Charuchaimontri et al., 1999; Chenthamarakshan et al., 2001). A monoclonal antibody (Mab Ps6F6) specific to *B. pseudomallei* exopolysaccharide offered significant protection in a murine sub-acute meliodosis model. The Mab Ps6F6 provided a moderate and transient reduction of inflammatory responses in infected mice but was only partially protection because this antibody was not able to provide sterilizing protective immunity (Bottex et al., 2005). Antibodies to *Burkholderia* Hep_Hag autotransporter (BuHA) proteins were present in horses with glanders and in human patients with melioidosis (Tiyawisutsri et al., 2007).

There is also evidence for an important protective role for B cells and antibodies in *Burkholderia* infection. For example, depletion of B cells significantly decreased survival time of *B. mallei*-infected BALB/c mice compared to non-depleted controls (Whitlock et al., 2008). It was speculated that antibody opsonization increased intracellular killing of *B. mallei* by increasing uptake by macrophages, but this was not formally demonstrated. Other studies have proposed that antibodies increase phagocytosis of *Burkholderia* (Bryan et al., 1994). Conversely, however, in a separate study when μMT mice (which lack all B cells) were infected with *B. mallei*, the B cell deficient animals were not found to be more susceptible to infection than wild type mice (Rowland et al., 2010). It is difficult to fully reconcile these very different results at present, but the discordant results could be a consequence of how B cells were depleted, of unanticipated effects of B cell depletion versus genetic deletion of B cells, or in the particular challenge models that were used in the studies.

## Targets for vaccination against *Burkholderia*

Currently, prolonged antibiotic therapy and early diagnosis are the only options for controlling infections with either *B. mallei* or *B. pseudomallei*. While *B. mallei* remain uncommon and largely controlled in the equine population, except in some Asian and African countries, *B. pseudomallei* is much more widely distributed worldwide (Blancou, 1994; Currie et al., 2008). In fact, recent studies point to an expanded range of *B. pseudomallei* endemic countries, including Brazil, Central America, and regions of Asia and India.

While these data do not necessarily suggest that *B. pseudomallei* is spreading, they do point out that a much greater population of civilians has some risk of contracting *B. pseudomallei* infection from their environment. However, the incidence of meliodosis does not currently justify vaccination, except in certain regions such as Thailand and northern Australia where the disease incidence is high enough to warrant consideration of a prophylactic vaccine. However, there are currently no approved vaccines for either meliodosis or glanders prevention in humans or animals. Thus, it is believed that efforts to develop a new *Burkholderia* vaccine can be readily justified. In the last portion of this review, we will discuss the various approaches that have been taken to produce effective *Burkholderia* vaccines. One substantial hurdle that must be overcome by *Burkholderia* vaccines is to protect from both acute infection and chronic infection. The latter is a much more difficult hurdle and few vaccines have demonstrated a convincing ability to fully protect from the development of the chronic infection phase of *B. pseudomallei* infection. Efforts to produce a *Burkholderia* vaccine can be broadly classified as subunit vaccines, live attenuated vaccines, and killed bacteria vaccines delivered with or without adjuvants. Table 1 summarizes several attempts to provide vaccine-mediated protection against glanders and melioidosis.

**Table 1:** **Melioidosis and glanders vaccine development**.

**Vaccine**	**Target bacterium**	**Route(s) of administration**	**Challenge model**	**Significant protection from challenge?**	**References**
Purified BipD protein	*B. pseudomallei*	i.p.^a^	*B. pseudomallei* strain K96243 by i.p.^a^ route	No	Stevens et al., 2004
BipB, BipC, and BipD protein	*B. pseudomallei*	i.p.^a^	*B. pseudomallei* Ashdown by i.p.^a^ route	No	Druar et al., 2008
Recombinant Hcp1, Hcp2, Hcp3, and Hcp6 protein	*B. pseudomallei*	i.p.^a^	*B. pseudomallei* K96243 by i.p.^a^ route	SNC^b^	Burtnick et al., 2011
Lipopolysaccharide (LPS)	*B. pseudomallei*	i.p.^a^	*B. pseudomallei* NCTC 4845T by i.p.^a^ and aerosol route	Partial protection for i.p.^a^ challenge No protection for aerosol challenge	Nelson et al., 2004
Monoclonal antibodies that recognize LPS	*B. pseudomallei*	i.p.^a^	*B. pseudomallei* NCTC 4845 by i.p.^a^ route	SNC^b^ and No protection with higher challenge dose	Jones et al., 2002
Purified flagellin	*P. pseudomallei*	i.p.^a^	*P. pseudomallei* 316c in diabetes rats by i.p.^a^ route	Partial protection in diabetes rats and Protection 4 out 5 (but 6 days after challenge)	Brett et al., 1994
LolC, PotF, and OppA-Non membrane protein	*B. pseudomallei*	s.c.^c^	*B. pseudomallei* K96243 and pseudomallei 576 by i.p.^a^ route	No protection or SNC^b^	Harland et al., 2007
Outer membrane vesicles	*B. pseudomallei*	s.c.^c^	*B. pseudomallei* 1026b by aerosol route	SNC^b^	Nieves et al., 2011
Recombinant Omp85 protein	*B. pseudomallei*	i.p.^a^	*B. pseudomallei* D286 by i.p.^a^ route	SNC^b^	Su et al., 2010
Live attenuated *B. pseudomallei purN, purM*, hisF, pabB mutant	*B. pseudomallei*	i.p.^a^ and i.n.^d^	*B. pseudomallei* E8 i.p.^a^, i.v.^e^, or i.n.^d^ route	SNC^b^ i.p.^a^ challenge; No protection to i.v.^e^ challenge	Breitbach et al., 2008
Live attenuated *B. pseudomallei* aroB mutant	*B. pseudomallei*	i.n.^d^	*B. pseudomallei* K96243	SNC^b^	Cuccui et al., 2007
Live attenuated *B. pseudomallei* 576 (ilvI2D2)	*B. pseudomallei*	i.p.^a^	*B. pseudomallei* 576 by i.p.^a^ route	SNC^b^	Atkins et al., 2002b; Haque et al., 2006a
Live attenuated *B. pseudomallei* bipD mutant	*B. pseudomallei*	i.p.^a^	*B. pseudomallei* K96243 by i.p.^a^ route	SNC^b^	Stevens et al., 2004
Whole cell *B. pseudomallei*	*B. pseudomallei*	s.c.^c^ and i.n.^d^	*B. pseudomallei* NCTC 13178 by i.v.^e^ route	No	Barnes and Ketheesan, 2007
Dendritic cells pulsed with heat killed *B. pseudomallei* K96243 and maturated in the presence of CpG 1826	*B. pseudomallei*	i.d.^f^	*B. pseudomallei* K96243, 576 and NCTC 4845 by i.p^a^ route	SNC^b^	Elvin et al., 2006
Whole killed *B. pseudomallei* adjuvant with CLDC	*B. pseudomallei*	i.n.^d^	*B. pseudomallei* 1026b by i.n^d^ route	SNC^b^	Henderson et al., 2011
BMA_A07682, BMA_28212, BMA_08162	*B. mallei*	i.m.^g^	*B. mallei* ATCC23344 by i.n.^d^ route	SNC^b^	Whitlock et al., 2011
Live attenuated *B. mallei* capsule mutant	*B. mallei*	aerosol	*B. mallei* ATCC 23344 by aerosol route	No	Ulrich et al., 2005
Live attenuated *B. mallei* branched-chain amino acid auxotroph mutant	*B. mallei*	aerosol	*B. mallei* ATCC23344 by aerosol route	SNC^b^	Ulrich et al., 2005
Whole killed *B. mallei*	*B. mallei*	i.p.^a^	*B. mallei* by i.p.^a^ and i.n.^d^ route	SNC^b^	Whitlock et al., 2008

a Intra peritoneal

b Signficant but not complete protection

c Subcutaneous

d Intranasal

e Intravenous

f Intraderm

g Intramuscular.

### Bacterial virulence factors as potential immune targets for vaccination

The ability to target bacterial virulence factors with vaccines has the potential to greatly attenuate the pathogenicity of *Burkholderia* and thereby suppress replication, allowing the host immune system to eradicate the organism before infection is well-established. A number of *Burkholderia* virulence factors have been identified and several have been evaluated as vaccine candidates.

#### Type III and VI secretion systems

*B. pseudomallei* type III secretion systems (T3SS) plays a role in early vacuolar escape, cell to cell spread in macrophages, and virulence (Stevens et al., 2002; Warawa and Woods, 2005; Burtnick et al., 2008). One component of the T3SS (BipD) has been tested as a candidate vaccine. Mice vaccinated by the i.p. route had a slightly prolonged early survival and reduced bacterial burden, but in general survival times were not prolonged compared to non-vaccinated mice subjected to the same *B. pseudomallei* challenge (Stevens et al., 2004). Other proteins from the T3SS system (BipB, BipC, or BipD) have also been evaluated as candidate vaccines, however, none of these three antigens generated protective immunity in challenge studies (Druar et al., 2008). In studies in our laboratory, we noted that intranasal vacctination of mice with BimA and two other *Burkholderia* antigens, induced protective immunity against an inhaled challenge (Whitlock et al., 2010).

The type VI secretion system (T6SS) is an important virulence factor that is necessary for the intracellular lifestyle of *B. pseudomallei*. Studies have shown that the T6SS Hcp1 protein from *B. pseudomallei* and *B. mallei* was recognized by convalescent serum from humans with documented meliodosis (Schell et al., 2007). Moreover, the T6SS proteins Hcp1, Hcp2, Hcp3, Hcp4, Hcp5, and Hcp6 have all been evaluated individually as candidate *B. pseudomallei* vaccines, but each has failed to provide complete protection in murine challenge models evaluated to date (Burtnick et al., 2011). However, it remains conceivable that a multi-valent T6SS vaccine could generate more effective immunity.

#### Quorum sensing molecules

Quorum sensing is important to the pathogencity of *Burkholderia*. In this process, molecules secreted by bacteria growing under conditions of crowding or stress help to regulate metabolism and survival functions of bacteria. LuxL and LuxR [*N*-acyl-homoserine lactone (AHL) receptors] are the most common signaling molecules found in gram negative bacteria and are also the major quorum sensing molecules involved in the virulence process of *Burkholderia* (Whitehead et al., 2001; Eberl, 2006). A *B. pseudomallei* LuxL mutant was significantly attenuated for infection in mice (Ulrich et al., 2004). The ability of several different *B. mallei* LuxI mutants to function as attenuated vaccines was also evaluated, using *bmaII, bmaI3*, and *bmaR5* mutants, followed by challenge with fully virulent *B. mallei*. Only mice vaccinated with the *bmaI3* mutant showed any level of protection, with survival of 3 of the 10 animals, 11 days after challenge (Ulrich et al., 2004).

#### Surface expressed molecules as vaccine targets

LPS and capsular polysaccharide (CPS) are the main surface-associated antigens of *B. mallei* and *B. pseudomallei* (Perry et al., 1995; Deshazer et al., 1998; Reckseidler et al., 2001; Atkins et al., 2002a; Sarkar-Tyson et al., 2007; Tuanyok et al., 2012). *B. pseudomallei* CPS is essential to bacterial virulence and this antigen activates the Th1 immune response (Warawa et al., 2009). Immunization against either antigen has been shown to provide partial protection against intranasal challenge with virulent *B. pseudomallei* (Trevino et al., 2006; Aucoin et al., 2012). The combination of both antigens provided better protection, even when administered in lower doses (Charuchaimontri et al., 1999; Jones et al., 2002; Nelson et al., 2004; Zhang et al., 2011). However, the antigen combination did not provide sterilizing immunity but did significantly decrease the bacterial burden in the spleen (Aucoin et al., 2012). In another study, vaccination with a mutant type II O-antigenic polysaccharide (O-PS) molecule also provided partial protection from infection with *B. pseudomallei* infection (Deshazer et al., 1998).

The efficacy of LPS or CPS as vaccines was tested in BALB/c mice (Nelson et al., 2004; Ngugi et al., 2010). Strong IgM and IgG3 responses were observed in mice vaccinated with LPS, whereas higher IgG2b responses were noted in mice immunized with CPS. After challenge with virulent *B. pseudomallei* by the i.p. route, there was partial protection in mice vaccinated with LPS, with 50% survival by day 35. Partial protection was also observed in the group vaccinated with CPS. However, when mice were challenged by the pulmonary route, there was no protection (Nelson et al., 2004). Thus, it appears that both LPS and CPS are important candidate vaccine antigens. Both antigens appear to provide at least partial protection from challenge with *Burkholderia* by parenteral routes, whereas neither has been particularly effective for protection from challenge by the inhalational route. In addition, neither LPS nor CPS vaccines protect from the development of chronic infection.

The ability of anti-LPS and anti-CPS monoclonal antibodies to provide passive protection from challenge with *Burkholderia* has also been evaluated. When mice were administered mAbs against LPS or CPS, there was protection against i.p. challenge with 10^4^ CFU of a virulent strain of *B. pseudomallei* (NCTC 4845) (Jones et al., 2002). However, at higher challenge doses, only a mixture of both mAbs was able to provide significant protection. In addition, the mAbs did not provide protection from aerosol challenge with *B. pseudomallei* (Nelson et al., 2004). Infusion of an anti-LPS antibody also protected mice from glanders when administered immediately prior to lethal challenge with *B. mallei* (Trevino et al., 2006).

#### Flagellin

Flagellin is an important molecule required for motility by *B. pseudomallei* (*B. mallei* does not have a flagellin molecule) (Deshazer et al., 1997; Wajanarogana et al., 1999; Chua et al., 2003; Nierman et al., 2004). In one study BALB/c mice were vaccinated with plasmid DNA encoding the flagellin gene *fliC*. Following intravenous challenge, 83% of the vaccinated mice survived for 7 days. Besides the increased survival rate, the vaccinated mice had a higher level of flagellin-specific antibodies and a higher level of IFN-γ production by splenocytes. It was suggested that the mechanism of protection was provided by the Th1 immune response due to the increased production of IFN-γ and the higher ratio of the IgG2a/IgG1 antibody response (Chen et al., 2006). Administration of a mAb directed against flagellin also provided partial protection (55%) to diabetic rats challenged by the i.p. route with *B. pseudomallei* (Brett et al., 1994). It was speculated that anti-flagellin antibodies may immobilize *Burkholderia* and thereby attenuate pathogenicity (Brett et al., 1994; Deshazer et al., 1997).

#### Outer membrane proteins

An outer membrane protein was evaluated as a meloidosis vaccine (Hara et al., 2009; Su et al., 2010). Immunization in BALB/c mice with outer membrane protein 85 (Omp85) using Freund's complete adjuvant, promoted a strong antibody responses and provided significant protection against *B. pseudomallei* D286 challenge with 70% of survival at 15 days post infection. However, sterilizing immunity was not achieved, as vaccinated mice developed chronic infection (Su et al., 2010).

### *Burkholderia pseudomallei* protein subunit vaccines

Several different immunogenic proteins of *Burkholderia* have been identified as potential candidate vaccine antigens. Protein subunit vaccines are appealing because vaccine production is simplified and with newer adjuvants many proteins can be rendered highly immunogenic.

#### Non-membrane proteins

Three different *B. pseudomallei* proteins from ATP-binding cassette (ABC) transport systems have been evaluated as potential vaccine candidates. One is LolC, which is an ABC transporter protein, first identified as an immunogen (Yakushi et al., 2000; Harland et al., 2007). The second antigen is PotF, which is a periplasmic binding protein of the PotFGHI system (Shah and Swiatlo, 2006) and is a homolog of the *Agrobacterium tumefaciens* PotF which is required for attachment and virulence (Matthysse et al., 1996). A third antigen (OppA), which is an oligopeptide-binding protein of the Opp system in *E. coli* (Detmers et al., 2001), has also been identified as a potential immunogen (Tanabe et al., 2006; Harland et al., 2007). Immunization with each of these proteins has been shown to stimulate antigen-specific humoral and cellular immune responses (Harland et al., 2007). Immunization with recombinant LolC or PotF proteins generated significant protection against *B. pseudomallei* challenge by the i.p. route and provided greater than 50% protection (Harland et al., 2007). In contrast, immunization with recombinant OppA protein did not generate significant protection. Since the LolC is also present in *B. mallei*, the authors speculated that LolC protein may also protect against glanders but this was not tested in the study (Harland et al., 2007).

In another study, immunogenic antigens were identified from a genomic screen of *B. mallei* expressed proteins. Three of these antigens, including LolC, were found to provide partial protection against *B. mallei* and *B. pseudomallei* following intranasal vaccination (Whitlock et al., 2010, 2011). Notably, the LolC antigen was shown to provide cross-protection against both *B. pseudomallei* and *B. mallei* in this intranasal vaccination model (Whitlock et al., 2010, 2011). Thus, it appears that protein subunit vaccines targeting highly immunogenic *Burkholderia* proteins have the potential to be used as effective vaccines. However, it is likely that polyvalent vaccines combining several antigens may be more effective than single antigen vaccines.

### *Burkholderia* live attenuated vaccines

A number of different live attenuated *Burkholderia* vaccines have been developed and evaluated in mouse challenge models. In general, live attenuated vaccines have been found to provide superior protective immunity when compared to other potential *Burkholderia* vaccines. However, the use of live attenuated vaccines by parenteral routes of immunization is associated with more safety and production issues compared with subunit vaccines.

#### *B. pseudomallei* live attenuated vaccines

Several different attenuated strains of *Burkholderia* have been evaluated as potential live vaccine strains for protection from *B. pseudomallei*. A number of different attenuation strategies have been investigated to produce these candidate vaccines.

The most extensively investigated live attenuated vaccine to date has been the 2D2, which is an auxotroph for branched chain amino acids. It is a mutant strain of *B. pseudomallei*, which was produced by mutating the *ilvI* gene encoding the large subunit of the acetolactate synthase enzyme (Atkins et al., 2002b). The 2D2 strain of *B. pseudomallei* was also shown not to persist *in vivo* following immunization (Atkins et al., 2002b). When BALB/c mice were vaccinated with 2D2 by the i.p route, there was significant but incomplete protection against i.p. challenge of fully virulent wild type *B. pseudomallei*. The incomplete protection was melioidosis specific since protection against *Francisella tularensis*, was not observed (Atkins et al., 2002b). The immune mechanism of protection appeared to require primarily CD4^+^ T cells, as CD4^+^ T cell depletion eliminated the protective effects of 2D2 vaccination (Haque et al., 2006a).

In another attenuated vaccine approach, a *B. pseudomallei* capsular mutant (*B. pseudomallei* 1E10) was evaluated as a vaccine candidate. Immunization with the capsular mutant 1E10 by the i.p. route resulted in no protection against virulent challenge (Atkins et al., 2002a).

Transposon mutants of *B. pseudomallei* have also been evaluated as candidate vaccines. Mutants in several key *Burkholderia* biosynthetic pathways (*purN, purM, hisF*, and *pabB*) have been investigated for protective immunogenicity (Pilatz et al., 2006; Breitbach et al., 2008). All of the mutant strains conferred some degree of protection against virulent challenge in susceptible BALB/c mice. Mice immunized by the intranasal and i.p. routes with the *purN* mutant were protected from acute infection following intranasal challenge (Breitbach et al., 2008). However, vaccinated mice were not protected from the development of chronic infection. The immunogenicity of a *purM* mutant strain of *B. pseudomallei* was also evaluated, but was found to provide only partial protection from virulent challenge, and immunization by either the intranasal or i.p. routes did not appear to affect protective efficacy (Breitbach et al., 2008). It was speculated that the greater apparent immunogenicity of the *purN* mutant bacterium was due to greater persistence of this strain following vaccination than the *purM* strain.

It should also be noted that a *purM* mutant strain (Bp82) of *B. pseudomallei* was recently approved by the CDC for use as an approved BSL2 level organism (Propst et al., 2010). This attenuated strain of *B. pseudomallei* was shown to be extremely safe even in very immune compromised animals.

*aroB* auxotrophic mutant strain 13B11 has also been evaluated as a candidate live attenuated vaccine (Cuccui et al., 2007). The 13B11 *aroB* mutant strain did not cause disease in mice when inoculated at either 1 × 10^5^ or 1 × 10^6^ CFU by the intranasal route. Furthermore, the *aroB* mutant was cleared from the lungs and spleen of mice within 3 days. Vaccinated mice showed an increased time to death when challenged with virulent *B. pseudomallei* but in the end all mice succumbed to the infection (Cuccui et al., 2007). An *aroC* deletion mutant of *B. pseudomallei* (A2) was also tested as a vaccine candidate (Foulongne et al., 2001; Srilunchang et al., 2009). Immunization with A2 by the i.p. route provided significant protection against virulent challenge in C57Bl/6 mice, but not in BALB/c mice (Srilunchang et al., 2009).

A T3SS apparatus mutant (*B. pseudomallei bipD*), has also been evaluated for protective immunity (Stevens et al., 2004; Druar et al., 2008). Mice inoculated with the *B. pseudomallei bipD* mutant strain were partially protected against subsequent challenge by the i.p. route with wild-type *B. pseudomallei*. In contrast, immunization of mice with recombinant BipD protein was not protective (Stevens et al., 2004; Druar et al., 2008).

#### *B. mallei* live attenuated vaccines

*B. mallei* capsule mutants and auxotrophs have been evaluated as glanders vaccines. Mice immunized by the aerosol route with a capsule mutant (DD3008) of *B. mallei* strain ATCC23344 developed high antibody responses (Ulrich et al., 2005). However, mice were not protected against aerosol challenge with a high dose of virulent *B. mallei*, presumably in part because the lack of mucosal IgA or IgG antibody responses (Ulrich et al., 2005). Immunization with an auxotrophic mutant of *B. mallei* (*ILV1*) elicited a Th1-like IgG subclass (IgG2a) antibody response, and provided significant short-term survival to challenge by the aerosol route with virulent *B. mallei*. In addition, immunization also provided significant protection to challenge by the aerosol route. It should be noted, however, that all mice surviving acute challenge developed chronic infection. It was speculated that the axotrophic mutants were more effective immunogens than capsule mutants (Amemiya, 2002; Ulrich et al., 2005). An attenuated *ctpA* mutant of *B. mallei* was also evaluated as a live attenuated vaccine, and found to provide partial protection against i.p. inoculation with virulent *B. mallei* (Bandara et al., 2008).

### Inactivated whole cell *Burkholderia* vaccines

Immunization with killed whole bacteria (bacterin vaccines) has been shown previously to provide effective immunization against a variety of bacterial diseases. These vaccines are relatively simple to produce and are safer than live attenuated vaccines and provide relatively high levels of specific immunity. However, vaccine site reactions are a potential problem with this class of vaccines.

#### *Burkholderia pseudomallei* killed vaccines

Live *B. pseudomallei* NCTC 13179, killed bacterial cells alone, or culture filtrate antigens (CFA) or lysates from *B. pseudomallei* cultures were tested as candidate vaccines following administration by the s.c. or i.v. routes, in BALB/c and C57BL/6 mouse i.v. challenge models. The live vaccine as well as lysates from *B. pseudomallei* and killed bacterial cells mixed with complete Freund's adjuvant all induced protective immunity. However, only the live vaccine stimulated a Th1 immune response (Barnes and Ketheesan, 2007).

In another study, the ability of vaccine adjuvants to enhance the immunogenicity of killed *Burkholderia* vaccines was evaluated. Mice were immunized intranasally with heat-killed *B. pseudomallei*, with or without a liposomal adjuvant (CLDC) and protective immunity to intranasal challenge assessed (Henderson et al., 2011). Immunization with CLDC adjuvant plus heat killed bacteria gave superior immunity compared to immunization with heat-killed bacteria alone.

The relative ability of heat-killed vaccines prepared with *Burkholderia thailandensis, B. mallei*, or *B. pseudomallei* cells was compared for induction of protection against melioidosis and glanders (Whitlock et al., 2008; Sarkar-Tyson et al., 2009; Henderson et al., 2011). Both the *B. mallei* and *B. pseudomallei* vaccines provided significant protection against *B. pseudomallei* challenge, whereas the *B. thailandensis* vaccine failed to elicit protective immunity. The probable mechanism of protection may have involved induction of antibody responses to the CPS and LPS and was CD4^+^ T cell dependent (Sarkar-Tyson et al., 2009).

#### *Burkholderia mallei* killed vaccines

Immunization with heat-killed *B. mallei* vaccines was evaluated for evidence of protection in mouse challenge models. Mice immunized i.p. with a heat-killed *B. mallei* vaccine were significantly protected from i.p. challenge with *B. mallei* (Whitlock et al., 2008). Antibody depletion studies indicated that both CD4^+^ T cells and B cells were required for induction of effective immunity following immunization with the heat killed vaccine, whereas CD8^+^ T cells appeared to be dispensable. Depletion of IFN-γ or TNF-α immediately prior to challenge also reduced vaccine effectiveness, suggesting a role for these cytokines in protective immunity.

#### Alternative vaccine approaches

Dendritic cell vectored vaccines have been shown to induce effective immunity against both pathogens and cancer. A DC vaccine prepared using DC pulsed *in vitro* with heat-killed *B. pseudomallei* was evaluated for effectiveness in a mouse *B. pseudomallei* challenge model (Elvin et al., 2006). Mice receiving the DC vaccine were significantly protected against challenge with virulent *B. pseudomallei* when delivered by the i.p. route.

Outer membrane vesicles (OMVs), which are secreted by bacteria during *in vitro* culture, have also been evaluated as candidate immunogens for *Burkholderia pseudomallei* and *mallei* (Hara et al., 2009; Nieves et al., 2011; Schell et al., 2011). Immunization with *B. pseudomallei* OMVs by the s.c. route was found recently to produce significant protection against pulmonary challenge with virulent *B. pseudomallei*. Induction of both cellular and humoral responses to vaccination was observed. Interestingly, immunization with OMVs by the intranasal route was not effective in generating protective immunity. OMV immunization also did not provide protection against chronic infection (Nieves et al., 2011).

The bacterial Elongation Factor-Tu (*EF-Tu*), which is also secreted in OMVs is immunogenic (Harding et al., 2007), and was evaluated as a vaccine antigen (Nieves et al., 2010). Immunization by the intranasal route resulted in reduced bacterial burden when mice were subjected to intranasal challenge with *B. thailandensis* (Nieves et al., 2010).

## Challenges facing *Burkholderia* vaccine development

While substantial progress has been made in the development of effective vaccines for *Burkholderia*, significant hurdles still remain. In the case of subunit vaccines, it is not clear whether new and more immunogenic target antigens have yet to be discovered. High throughput screening strategies may facilitate the identification of such antigens. In addition, the development of new polyvalent vaccines may provide more effective and broader coverage against *B. pseudomallei* infection than single subunit vaccines, which is particularly important given the high genetic diversity of different *B. pseudomallei* isolates, even from the same patients.

It will also be important to more fully define the correlates of protective immunity to *Burkholderia*. For example, given that CD4^+^ T cells are important in protective immunity, are certain CD4^+^ T cell functional subsets (eg, Th17, Th2, or Th1 cells) more desirable than others in generating strong and long-lasting protective humoral and cellular immune responses? It is also likely that the role of CD8^+^ T cells may be more important than early studies have suggested, particularly for prevention of the chronic stage of infection.

Given that chronic *Burkholderia* infection is likely contracted via mucosal routes of exposure to *Burkholderia*, development of vaccines that generate strong mucosal immune responses and IgA production is an important goal. This will necessitate the use of challenge models that mimic natural route of exposure, including oral and inhalational exposure, as well as cutaneous inoculation. Newer vaccine adjuvants may be particularly effective in development of strong humoral or cellular immune responses, or more balanced responses, depending on what is required for protective immunity. The route of immunization is also an important variable, particularly for vaccines designed to provide protection from pulmonary or oral challenge with *Burkholderia*.

Is there a future for a live attenuated *Burkholderia* vaccine? At present, these vaccines are the most likely to generate full protective immunity against a broad range of *Burkholderia* strains, using relatively simple immunization protocols. Whether these vaccines will be adopted will depend on the target population for the intended vaccine and the willingness of local regulatory authorities to approve live attenuated vaccines. Full demonstration of vaccine safety and efficacy against a variety of different challenge strains and routes can help advance the case for live attenuated *Burkholderia* vaccines. Finally, while a number of candidate vaccines have demonstrated an ability to protect from acute challenge with *Burkholderia*, it is not currently possible to provide effective protection against the development of chronic *Burkholderia* infection. Thus, greater attention to development of effective mucosal *Burkholderia* vaccines that are capable of generating sterilizing immunity is warranted in the future.

### Conflict of interest statement

The authors declare that the research was conducted in the absence of any commercial or financial relationships that could be construed as a potential conflict of interest.
